# Cavity Floquet engineering

**DOI:** 10.1038/s41467-024-52014-0

**Published:** 2024-09-06

**Authors:** Lingxiao Zhou, Bin Liu, Yuze Liu, Yang Lu, Qiuyang Li, Xin Xie, Nathanial Lydick, Ruofan Hao, Chenxi Liu, Kenji Watanabe, Takashi Taniguchi, Yu-Hsun Chou, Stephen R. Forrest, Hui Deng

**Affiliations:** 1 Department of Physics, University of Michigan, 450 Church Street, Ann Arbor, MI 48109-2122 USA; 2 Department of Electrical Engineering and Computer Science, University of Michigan, 1301 Beal Avenue, Ann Arbor, MI 48109-2122 USA; 3 Applied Physics Program, University of Michigan, 450 Church Street, Ann Arbor, MI 48109-2122 USA; 4 Nuclear Engineering and Radiological Science, University of Michigan, 2355 Bonisteel Blvd, Ann Arbor, MI 48109-2122 USA; 5 Research Center for Electronic and Optical Materials, National Institute for Materials Science, 1-1 Namiki, Tsukuba, 305-0044 Japan; 6 Research Center for Materials Nanoarchitectonics, National Institute for Materials Science, 1-1 Namiki, Tsukuba, 305-0044 Japan; 7 Department of Photonics, National Cheng Kung University, Tainan, ROC Taiwan; 8 Academy of Innovative Semiconductor and Sustainable Manufacturing, National Cheng Kung University, Tainan, ROC Taiwan

**Keywords:** Nonlinear optics, Two-dimensional materials, Photonic devices

## Abstract

Floquet engineering is a promising tool to manipulate quantum systems coherently. A well-known example is the optical Stark effect, which has been used for optical trapping of atoms and breaking time-reversal symmetry in solids. However, as a coherent nonlinear optical effect, Floquet engineering typically requires high field intensities obtained in ultrafast pulses, severely limiting its use. Here, we demonstrate using cavity engineering of the vacuum modes to achieve orders-of-magnitude enhancement of the effective Floquet field, enabling Floquet effects at an extremely low fluence of 450 photons/*μ*m^2^. At higher fluences, the cavity-enhanced Floquet effects lead to 50 meV spin and valley splitting of WSe_2_ excitons, corresponding to an enormous time-reversal breaking, non-Maxwellian magnetic field of over 200 T. Utilizing such an optically controlled effective magnetic field, we demonstrate an ultrafast, picojoule chirality XOR gate. These results suggest that cavity-enhanced Floquet engineering may enable the creation of steady-state or quasi-equilibrium Floquet bands, strongly non-perturbative modifications of materials beyond the reach of other means, and application of Floquet engineering to a wide range of materials and applications.

## Abstract (web summary)

The authors demonstrate enhancement by nearly two-orders of magnitude of the optical Stark effect in WSe_2_ embedded into a Fabry Perot cavity, and use this mechanism to obtain an effective magnetic field of over 200 T and implement a XOR switch.

## Introduction

Coherent electromagnetic waves form a periodic potential in time for electric dipoles and modify electronic transitions, which is known as Floquet engineering^1–3^. By controlling the frequency and spatial-temporal mode of an off-resonant laser field, different Floquet potentials can be formed to modify the spatial-temporal symmetry^4–7^, topology^8–10^, and energy landscape^11–13^ of electronic transitions, potentially rendering rich new phenomena and novel applications^14,15^. In most materials, however, Floquet effects are often overwhelmed by inhomogeneity, phonon-induced dephasing, and other dissipation channels. Ultrafast lasers with high fluence are typically required to produce significant Floquet effects. This has restricted the application of Floquet engineering to a few materials and to transient phenomena that can be difficult to model, understand, or use. To circumvent complications of high-intensity lasers, vacuum fields in cavities have been explored in theory for Floquet engineering recently^16,17^. Alternatively, cavities can also be designed to modify the effective field of a driving laser and thus the Floquet effects^18,19^. Here we demonstrate the use of an optical cavity to achieve two orders of magnitude enhancement of the effective fluence of a driving Floquet field, enabling an enormous non-Maxwellian magnetic field over 200 T and an ultrafast, picojoule all-optical-chirality XOR gate. Our results also suggest that a sufficiently large static effective magnetic field and steady-state Floquet engineering can be achieved with commonly available continuous-wave lasers. The work opens doors to Floquet engineering of a wide range of materials, frequency bands, and spatio-temporal mode structures.

**Fig. 1:**
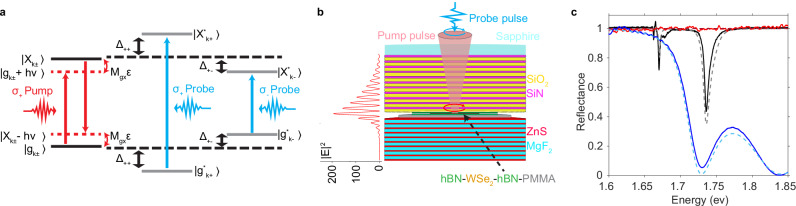
The principle and experimental system of cavity-enhanced optical Stark effect (OSE). **a** Illustration of the red-detuned chiral OSE. The circularly polarized pump leads to Floquet states ($\left\vert {{\rm{X}}}-{{\rm{h}}}\nu \right\rangle$ and $\left\vert {{\rm{g}}}+{{\rm{h}}}\nu \right\rangle$), which hybridize with the ground and excited state of excitons $\left\vert {{\rm{g}}}\right\rangle$ and $\left\vert {{\rm{X}}}\right\rangle$, resulting in blueshifted dressed states $\left\vert {{{\rm{g}}}}^{{\prime} }\right\rangle$ and $\left\vert {{{\rm{X}}}}^{{\prime} }\right\rangle$. A weak probe pulse measures the shifted transition energy. **b** Schematic of the half-wavelength cavity with a monolayer WSe_2_ at the antinode. The simulated field distribution at the cavity resonance is plotted on the left side, showing a 200-fold enhancement of the resonant field at the antinode. Optical measurements are performed through transparent Sapphire. **c** Reflectance spectra of the ${{\rm{SiN}}}/{{{\rm{SiO}}}}_{2}$ bottom distributed Bragg reflector (DBR) (blue) with a side-band minimum at the exciton resonance, the ZnS/MgF_2_ top DBR (red) with high reflectance at the laser and exciton energies, and the complete cavity (black) showing cavity (1.67 eV) and exciton (1.74 eV) resonances. The solid/dashed curves are the measured/simulated results, respectively.

To demonstrate cavity-enhanced Floquet engineering, we consider the Floquet effects of a pulsed driving field on excitonic transitions (Fig. 1a), which is also known as the optical Stark effect (OSE)^20,21^. OSE has been used to create optical trapping potentials for ultracold atomic gases, where even a small shift in resonance is pronounced compared to the narrow linewidths of atomic transitions^22–24^. In materials, OSE has been studied since the 1980s^25,26^ and, more recently, in transition metal dichalcogenide (TMD) monolayers^4–6,27^. Yet the change of the resonances due to non-resonant OSE has been less than their linewidths, leaving the effects in the perturbative regime. Lasers resonantly driving discrete and continuum electronic transitions can produce strong modifications to materials, yet absorption-induced incoherent effects and multitudes of higher-order effects often dominate^13,28–30^.

In this work, we demonstrate coherent, non-resonant OSE well beyond the perturbative regime via cavity enhancement. We measure a shift of the exciton transition energy up to 46 meV in a WSe_2_ monolayer, many times the inhomogeneously broadened exciton linewidth of 10 meV, and a valley splitting of 50 meV, corresponding to a non-Maxwellian magnetic field of  >200 T^31,32^.

## Results

### The system

Our system can be modeled by a four-level Hamiltonian in the field interaction picture. As illustrated in Fig. 1a, we consider a laser field with frequency *ν* and amplitude *ε*, coupled to the exciton transition from the ground state of the material $\left\vert g\right\rangle$ to the exciton state $\left\vert X\right\rangle$, with a transition matrix element *μ*_*gX*_ (Fig. 1a). The exciton energy is *E*_*X*_, and the laser is red-detuned from *E*_*X*_ by Δ  = *E*_*X*_  −  *hν*. In the field interaction picture, the exciton-field coupling leads to two virtual states $\left\vert X-h\nu \right\rangle$ and $\left\vert g+h\nu \right\rangle$, coupled with $\left\vert g\right\rangle$ and $\left\vert X\right\rangle$ respectively, with a coupling strength *μ*_*gX*_∣*ε*∣ (Fig. 1a). Using base vectors $\left\vert g\right\rangle,\left\vert X-h\nu \right\rangle,\left\vert g+h\nu \right\rangle$ and $\left\vert X\right\rangle$, the system Hamiltonian is given by:

1 $$H=\left(\begin{array}{cccc}0&{\mu }_{gX}| \varepsilon | &0&0\\ {\mu }_{gX}| \varepsilon | &\Delta &0&0\\ 0&0&{E}_{X}-\Delta &{\mu }_{gX}| \varepsilon | \\ 0&0&{\mu }_{gX}| \varepsilon | &{E}_{X}\\ \end{array}\right)$$

Diagonalizing the Hamiltonian gives the energies of the shifted energy levels $\left\vert {g}^{{\prime} }\right\rangle$ and $\left\vert {X}^{{\prime} }\right\rangle$, and thus the dressed-exciton energy *E*_*X*_  +  Δ*E*, where the Stark shift Δ*E* is given by:

2 $$\Delta E=-\Delta+\sqrt{{\Delta }^{2}+4{\mu }_{gX}^{2}| \varepsilon {| }^{2}}.$$

Under the weak field approximation, ${\mu }_{gX}^{2}| \varepsilon {| }^{2}/{\Delta }^{2} \, \ll \, 1$, the Stark shift reduces to $\Delta E\cong 2{\mu }_{gX}^{2}| \varepsilon {| }^{2}/\Delta,$ which increases linearly with the field intensity ∣*ε*∣^2^. In a resonant cavity, the effective field intensity at the central antinode is enhanced by a factor ${\eta }_{cav}\equiv | \varepsilon {| }^{2}/| {\varepsilon }_{in}{| }^{2} \sim {{\mathcal{F}}}/\pi$, where ${{\mathcal{F}}}$ is the finesse of the cavity, and *ε*_in_ is the input field amplitude in free space. The enhancement *η*_cav_ can be many orders of magnitude in typical microcavities.

To achieve optimal cavity enhancement for a red-detuned pump, we design an asymmetric *λ*/2 cavity as illustrated in Fig. 1b. A higher quality factor would produce a stronger enhancement, but only for frequencies within the cavity linewidth. Therefore, we match the resonance frequency and linewidth of the cavity with those of our 360-fs pulsed pump laser with 2.3 meV linewidth, 67 meV red detuning from the WSe_2_ A exciton resonance. The enhancement factor averaged over the pulse is calculated to be 145. To facilitate probing the exciton transition, the first side-band minimum of the top mirror is positioned at the exciton frequency, while the bottom mirror has a stopband that covers both the pump laser and exciton frequencies, as shown in Fig. 1c. (See Methods for more details).

To study the OSE of our device, we use an ultrafast pump pulse and measure the change induced by the pump via the reflectance contrast (RC) spectra of a weak white-light probe (Fig. 1b). The pump and probe pulses are both 360 fs in duration with variable relative delay. Due to spin-valley locking in TMDs^33–36^, the chirality of the pump and probe allows valley selectivity.

### Cavity enhancement

**Fig. 2:**
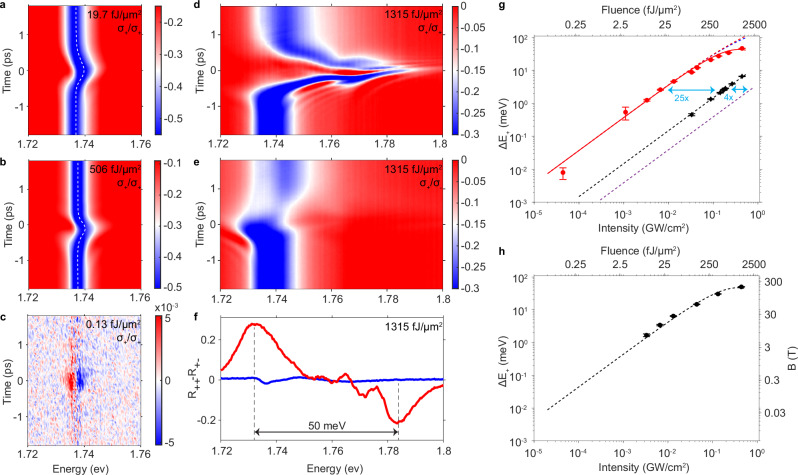
Extreme Floquet engineering. **a**, **b** Co-circularly polarized probe reflectance spectra *R*_++_(*t*) at different delay time *t* relative to the pump. **a** The monolayer is in a cavity, and pump fluence P = 18 fJ/μm^2^. **b** The monolayer is on a DBR, and P = 460 fJ/μm^2^. The dashed white curves are fitted exciton resonances. The same amount of Stark shift of  ~2.5 meV is observed when the pump and probe overlap in time, while the pump fluences differ by 26 times between the cavity and DBR devices. **c** Weak pump, co-circular differential reflectance spectra Δ*R*_++_(*t*)/*R*_++_(*t*) for the cavity device with a pump intensity of 33 kW/cm^2^, or a fluence of 0.12 fJ/μm^2^. **d** Strong pump, co-circular reflectance spectra *R*_++_(*t*) of the cavity device with a pump intensity of 330 MW/cm^2^ or fluence of 1.2 pJ/μm^2^, showing a very large blueshift at zero time delay. **e** Cross-circular probe *R*_+−_(*t*) under the same pump as in (**d**), showing a redshift at zero time delay. **f** Energy difference between *K* and ${K}^{{\prime} }$ valley excitons when the same pump as in (**d**, **e**) is turned on (red) and off (blue) at time = 0, showing effective Zeeman splitting of 50 meV. **g** Co-circular Stark shift vs. the pump fluence and intensity for a monolayer in a cavity (red), on a DBR (black), and in free space (purple). Symbols are measurement results. The red and black dashed lines are fits using Eq. (2). The red solid line is a fit including both two-photon absorption and cavity-shifting. The blue dashed line is a fit only with cavity-shifting. The purple dashed line is an extrapolation from DBR to vacuum. **h** Dressing induced valley splitting vs. the pump fluence and Intensity for the cavity device. Symbols are measurement results. The black dashed line is model prediction.

We first measure the cavity enhancement by comparing the pump laser intensity required for the same optical Stark shift in the cavity versus on a TiO_2_/SiO_2_ mirror. The mirror is designed to have a high-reflectance stopband that covers both the cavity and exciton energies (see Supplement Materials). As shown in Fig. 2, similar blueshifts of exciton absorption, Δ*E*, are clearly seen in the RC spectra when the WSe_2_ monolayer is either placed inside a cavity (Fig. 2a) or on a mirror only (Fig. 2b), with fitted Δ*E*  =  2.58  ±  0.09 meV and 2.52  ±  0.07 meV, respectively. Blueshifts appear only when the pump and probe pulses overlap in time, which confirms that they result from the OSE. However, the pump pulse fluence used is only 18 fJ/μm^2^ when in the cavity (Fig. 2a), compared to 460 fJ/μm^2^ when on the Distributed Bragg reflector (DBR) (Fig. 2b). Since Δ*E*/Δ ≈2.5/67 ≈ 0.037 ≪ 1, the weak field approximation is valid. Using Δ*E* ∝ ∣*ε*∣^2^, the cavity enhances the effective field intensity by 26-fold compared to a DBR. Since the DBR already provides a 3.4-fold enhancement of effective field intensity relative to free space (see Supplement), we deduce a cavity enhancement factor *η*_cav_  =  88.

Fitting the dependence of Δ*E* on pump intensity in the weak field limit (*P* <  0.015 GW/cm^2^) with Eq. (2), we obtain ${\mu }_{gX}^{2}\cdot {\eta }_{cav} \, \approx \, 4900\pm 100\,{{\mbox{Debye}}}^{2}$ (red dashed line in Fig. 2g). Comparing this value with ${\mu }_{gX}^{2}$ of 59 Debye^2^ ^37^ and 45 Debye ^2^^4^ previously measured by absorption and OSE, we obtain *η*_cav_ ≈83 to 109, consistent with the measured value of 88.

### Pump fluence dependence of enhanced OSE

The large cavity enhancement allows OSE in regimes that were difficult to access previously. As an example in the weak-field limit, Fig. 2c shows where the Stark shift remains clearly discernible down to an extremely low pump fluence of 0.12 fJ/μm^2^, or about 450 photons/μm^2^. The corresponding intensity is 0.33 mW/μm^2^ during our 360 fs pulse. This suggests that the effect is achievable using a continuous-wave (CW) laser of only 5.6 mW power over 17 μm^2^. In contrast, in free space, a high power of about 500 mW would be required for CW OSE.

The cavity enhancement also allows us to reach an abnormally large coherent Stark shift beyond the weak-field approximation, even with a large pump detuning. Figure 2d shows an example where a Stark shift of 46.3  ±  0.4 meV is measured for Δ =  67 meV. This is the highest reported coherent optical Stark shift in TMD.

In Fig. 2g, we summarize the pump intensity dependence of the cavity-enhanced Floquet. The weak-field, linear regime extends from an extremely low pump fluence of 0.12 fJ/μm^2^ to about 40 fJ/μm^2^, with corresponding Stark shifts of 0.008 meV to about 4.6 meV. Throughout this regime, we observe the same 26-fold enhancement of the Stark shift in the cavity (red circles in Fig. 2g) compared to the DBR (black diamonds), or equivalently 88-fold compared to free space (purple dashed line). For a pump fluence  >100 fJ/μm^2^, the Stark shift deviates from the predictions of Eq. (2) (red dashed line in Fig. 2g), and eventually saturates to a maximum shift of about 50 meV. We will discuss later possible causes of the saturation and mitigation strategies.

### Ultrahigh effective magnetic field

The cavity-enhanced Floquet effect can be used to create a non-Maxwellian magnetic field B* in the material based on the optical selection rules of exciton-photon coupling. In TMDs, optical selection rules lead to valley selectivity due to spin-valley locking. We use probe pulses that are co- or cross-circularly polarized with the pump to separately measure the OSE of the two valleys. As shown in Fig. 2d–f, a 1200 fJ/μm^2^*σ*_+_ pump introduces a blueshift of 46.3  ±  0.4 meV of the co-circular OSE, but a redshift of 3.8  ±  0.2 meV for the cross-circular OSE. The latter has been attributed to many-body Coulomb interactions, with biexciton as the dominant contribution^38–40^. Therefore, the 1200 fJ/μm^2^ pump pulse leads to a spin and valley splitting of Δ_*Z*_  =  50 meV in WSe_2_ exciton. Using the exciton g factor of 1.9 in an early report^41^, the same amount of Zeeman splitting would require an external magnetic field of *B* ~ 455 T. Using the g factor of 4.1 in more recent work^31^ would give *B* ~211 T. Figure 2h shows the control of B* by pump fluence.

### Ultrafast low-power chirality XOR switch

Lastly, we show that the enhanced OSE may facilitate energy-efficient all-optical computing. A switch, which functions as an XOR gate, is a critical building block for optical computing. A coherent optical switch has the advantage of ultrafast speed and minimal pump-induced heating. While coherent OSE can change the optical response of the system on an ultrafast timescale, it has been impractical for switching when it has required high-intensity lasers and produced only weak responses. In our cavity device, however, a large optical Stark shift can be achieved using a low-intensity pulse with minimal excitation of incoherent carriers.

**Fig. 3:**
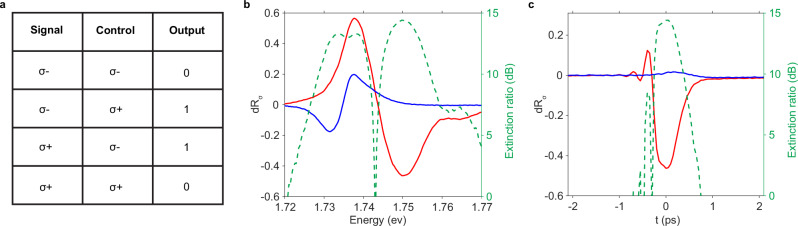
A chirality all-optical switch. **a** The truth table of an XOR gate. When control is co-circular to the signal, a decrease in the reflected signal is defined as “0” output. When control is cross-circular to the signal, an increase in the reflected signal is defined as “1” output. **b** Left axis: The modulation amplitude of the probe reflectance by a 120 fJ/μm^2^ (2 pJ) pump at zero time delay: *dR*_±_  = *R*_±_(on) −  *R*_±_(off), where  ± denotes the co- (blue) and cross-circularly (red) polarized probe. Right axis: The corresponding extinction ratio *Θ*  = 10*log*_10_(∣*dR*_+_  −  *dR*_−_∣/*δ*) (green). **c** Time-dependence of the modulation amplitudes for the co- (blue) and cross-polarized (red) probe and the corresponding extinction ratio *Θ* (green) at E = 1.75 eV, showing an ultrafast switch time of about 0.4 ps, reflecting the coherent nature of the Floquet effect.

As an example, we evaluate our device as a chirality XOR gate. A chirality XOR is a logic gate with the additional chirality degree of freedom, where the output signal is logic 1/0 according to the chirality of the two input beams (Fig. 3a). In TMDs, chirality is also locked with the valley degree of freedom. With a 120 fJ/μm^2^ (2 pJ) pump, we obtain a valley splitting of 14.6  ±  0.3 meV (~1.5 × linewidth) in the WSe_2_ exciton. Figure 3b shows the corresponding change in normalized reflectance *dR*_*σ*_  =  *RC*_pump−on_  − *RC*_pump−off_, where *σ*  =  +( − ) presents the co- (cross-) circularly polarized probe. Since it is straightforward to add a non-dispersive linear absorptive medium to uniformly reduce the minimum reflectance, we consider the extinction ratio of the switch as the contrast between *σ* ± vs. the power noise *δ*: $10{\log }_{10}(| {{{\rm{dR}}}}_{+}-{{{\rm{dR}}}}_{-}| /\delta )$. From the variance of 500 pulses, we obtain *δ* ~ 1.1− 2.0%. The extinction ratio is shown in Fig. 3b (right axis), which reaches about 15 dB at *E* ~ 1.75 eV. Furthermore, as a coherent switch, the switching has the same ultrafast bandwidth as the pump. Figure 3c shows the time-dependent modulation amplitude at *E*  =  1.75 eV. A swap of pump and probe polarization will complete the truth table of an XOR gate listed in Fig. 3a. Coherent switching with similar speed and power has only been achieved using 6-mm LiNbO_3_ bulk crystals^42^. The performance of our switch and XOR gates can be further improved by reducing the inhomogeneously broadened exciton linewidth.

## Discussion

The demonstrated time-reversal breaking, manifested in valley splitting of 50 meV, is already beyond the reach of presently available Maxwellian magnetic fields. An intriguing question is how strong the effect, or, how large an optical Stark shift Δ*E*, can be reached through cavity-enhanced Floquet engineering.

We consider two main factors that lead to the saturation of Δ*E* in our present experiment. First, the large shift in the exciton resonance leads to a change in the dielectric constant of the monolayer, which in turn shifts the cavity resonance and reduces the enhancement factor *η*_*cav*_ at the original cavity frequency. For our device, we calculate a reduction of *η*_cav_ by up to 13% at the pump fluence used, shown by the blue dashed line in Fig. 2g (See Supplement). However, this is not an intrinsic limitation and can be mitigated by tuning the pump laser wavelength to match the shifted cavity resonance.

Second, linear and higher-order absorption of the red-detuned pump becomes non-negligible at high pump intensities, leading to excitations in the monolayer, which saturate the exciton transition dipole moment *μ*_*gX*_. This can be seen in Fig. 2d, e, where the exciton resonance shows reduced reflection contrast, as well as a small blueshift after the pump. We measure the excitations generated by the pump through time-integrated photoluminescence (PL), which shows that linear and two-photon absorption (TPA) are the dominant contributions, and TPA dominates above *P* ~ 0.2 GW/cm^2^ (see Supplement). Including both the shift of the cavity resonance and the saturation of *μ*_*gX*_, we achieve excellent agreement between the measured and fitted pump intensity dependence of the optical Stark shift (the red symbols and red solid line in Fig. 2g).

The above analysis suggests that the maximum shift can be increased in a few ways. First, we can blueshift the pump to follow the blueshift of the cavity resonance, typically by less than 1 meV adjustment. Second, linear absorption can be strongly suppressed by decreasing the exciton linewidth toward the radiative limit, or by increasing the red detuning, Δ, of the pump. Although the Stark shift is also reduced inversely with Δ, it can be countered by a linear increase in pump intensity until TPA dominates. The main limitation comes from TPA, as its efficiency is approximately constant for detuning much less than half of the exciton resonance^43^.

**Fig. 4:**
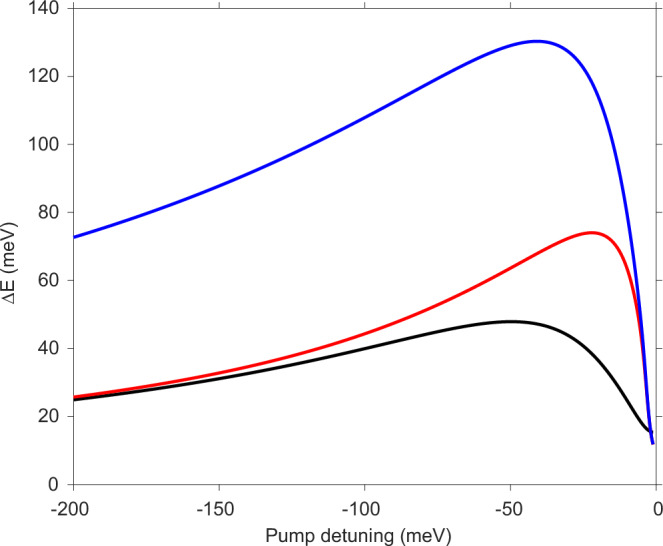
Optimal Floquet shift. Calculated saturated Floquet shift vs. the pump detuning for our current device (black), a device with 1 meV exciton linewidth (red), and a device with 1 meV exciton linewidth and an order of magnitude smaller TPA (blue). Other parameters are the same as the current device.

We evaluate possible maximum shifts using the fitted linear and TPA coefficients for our device. The exciton is modeled as a Lorentz oscillator, and the pump as a Fourier-limited pulse with a Gaussian spectrum (see Supplement for details). As shown in Fig. 4, we calculate a maximum optical Stark shift of Δ*E*_max_ ~ 48 meV for our device at an optimized pump red detuning Δ_opt_ ~ 49 meV. Considering an exciton linewidth of 1 meV, which is close to the radiative limit, we obtain Δ*E*_max_ ~ 74 meV with Δ_opt_ ~ 22 meV. If TPA can be suppressed tenfold, then Δ_opt_ ~ 41 meV leads to Δ*E*_max_ ~ 131 meV.

In conclusion, we demonstrate near two orders of magnitude enhancement of the effective Floquet field intensity in an asymmetric cavity compared to in free space. This approach has enabled a valley splitting as large as 50 meV that corresponds to time-reversal breaking by a 210 T magnetic field, a measurable Floquet effect with a pump fluence as low as 0.12 fJ/μm^2^ or an average intensity of 33 kW/cm^2^, and an ultrafast coherent chirality XOR switch with 15 dB on/off switching ratio. These effects can be further enhanced by reducing the exciton linewidth to the radiative limit and suppressing high-order nonlinear processes.

Theory work suggests a corresponding enhancement of a Maxwellian magnetic field via the inverse Faraday effect^32^. Future work may identify the optically-induced Maxwellian magnetic field, and may clarify the nature of the effective non-Maxwellian magnetic field we have measured here, such as its effects on the electronic band structures and dielectric breakdown of materials in contrast to a Maxwellian magnetic field.

The demonstrated cavity-enhanced Floquet can be broadly applied to optically active materials of different spectral bands to induce pseudo-magnetic fields inaccessible by other means, to enable CW Floquet engineering of new quantum phases^44^, and to facilitate ultra-low-energy, ultrafast, all-optical switches and sensors^45^. In materials with stronger light-matter interactions, cavities have been explored as an alternative to laser driving for Floquet engineering^16,17^.

## Methods

### Composition and fabrication of the device

The device consists of, from substrate to top, a bottom DBR, a WSe_2_ monolayer encapsulated by hexagonal boron nitride (hBN) crystals, a polymethyl methacrylate (PMMA) spacer layer, and a top DBR. The bottom DBR comprising 10.5 pairs of ${{\rm{SiN}}}$ (~102 nm)/SiO_2_ (~138 nm) and 70-nm top spacer SiO_2_ layers are grown on 500-μm thick Sapphire substrate by plasma enhanced chemical vapor deposition (PECVD). The monolayer WSe_2_ and two few-layer hBN flakes are mechanically exfoliated from bulk crystals. A polypropylene carbonate (PPC) film combined with polycarbonate (PC) protrudes, and a polydimethylsiloxane (PDMS) stamp is used to pick up the top hBN, WSe_2_ monolayer, and the bottom hBN under a microscope. After all of the layers are picked up, the PPC film is melted, stamped onto the bottom DBR, and then cleaned in dichloromethane. After the monolayer is placed on the bottom DBR, a PMMA layer is spin-coated on top, followed by the transfer of the top DBR^46^. The top transferable DBR comprising 9.5 pairs of ZnS (~75 nm) and MgF_2_ (~123 nm) layers is grown on a silica substrate by thermal evaporation in a vacuum chamber with a base pressure of 10^−7^ Torr. The transferable DBR allows us to form the cavity with precise control of the cavity resonance without degrading the 2D materials^46^.

### Circularly-polarized pump-probe spectroscopy

A 360-fs pump pulse with tunable photon energy is generated from a high-power 1035-nm pulse at a 100-kHz repetition rate through an optical parametric amplifier (OPA) and filtered to 2.3 meV linewidth by tunable spectral filters. The probe pulse is a 1.2 ps chirped white light supercontinuum with a spectral range of 645–735 nm, generated by focusing the 1035-nm residue pulse from the OPA onto a YAG crystal. We use a linear chirp correction for the probe. A motorized delay stage controls the pump-probe time delay. The pump beam is chopped at 30 Hz and focused to a waist size of 2.3 μm on the sample. The probe is focused on a slightly smaller beam at the center of the pump. The total fluence of the probe is 54.8 fJ/μm^2^; the fluence at the exciton resonance is about 0.6 fJ/μm^2^/nm, and there is no cavity enhancement at the exciton resonance. The probe-induced shift or saturation of the exciton resonance is observed. The pump-on/off intensities of the reflected probe are recorded by a spectrometer, synchronized with the chopper at double its frequency (60 Hz). The circular polarizations of the pump and probe are independently controlled by quarter-wave and half-wave plates and linear polarizers.

We probe the valley-selective OSE via circularly polarized pump-probe spectroscopy. Due to TMD spin-valley lock-in^33–36^, a co-circularly polarized pump and probe will dress and detect the exciton in the same valley, while cross-circular polarization means dressing ${{\rm{K}}}/{{{\rm{K}}}}^{{\prime} }$ valley but detecting ${{{\rm{K}}}}^{{\prime} }/{{\rm{K}}}$ valley.

### TMM simulation

We use the transfer matrix method (TMM) to calculate the device’s reflection spectrum and the electric field distribution of the cavity device. The simulated refractive index of the materials: ${{{\rm{SiO}}}}_{2},\,{{\rm{SiN}}},\, {{{\rm{MgF}}}}_{2},\,{{\rm{ZnS}}},\,{{{\rm{TiO}}}}_{2}$ are 1.47, 2.00, 2.32, 1.42, and 2.53, respectively.

## Supplementary information

Supplementary Information

Peer Review File

## Data Availability

Source data generated in this study is available at the repository DeepBlue under 10.7302/f6xv-d389.
