# Supplementary material for: Cavity Floquet engineering
Source: Nat Commun. 2024 Sep 6;15:7782. doi: 10.1038/s41467-024-52014-0 (PMC11377740; doi:10.1038/s41467-024-52014-0)
Supplement: Supplementary file 1 — Supplementary Information [file 41467_2024_52014_MOESM1_ESM.pdf]

**SUPPLEMENTAL MATERIALS**

# CAVITY FLOQUET ENGINEERING


Lingxiao Zhou[1], Bin Liu[2], Yuze Liu[2], Yang Lu[2], Qiuyang Li[1], Xin Xie[1], Nathanial Lydick[1], Ruofan Hao[3], Chenxi Liu[4], Kenji Watanabe[5], Takashi Taniguchi[6], Yu-Hsun Chou[7,8], Stephen R. Forrest[1,2] and Hui Deng[1,2]

[1] *Department of Physics, University of Michigan, 450 Church Street, Ann Arbor, MI 48109-2122, USA*

[2] *Department of Electrical Engineering and Computer Science, University of Michigan, 1301 Beal Avenue, Ann Arbor, MI 48109-2122, USA*

[3] *Applied Physics Program, University of Michigan, 450 Church Street, Ann Arbor, MI 48109-2122, USA*

[4] *Nuclear Engineering and Radiological Science, University of Michigan, 2355 Bonisteel Blvd, Ann Arbor, MI 48109-2122, USA*

[5] *Research Center for Electronic and Optical Materials, National Institute for Materials Science, 1-1 Namiki, Tsukuba 305-0044, Japan*

[6] *Research Center for Materials Nanoarchitectonics, National Institute for Materials Science, 1-1 Namiki, Tsukuba 305-0044, Japan*

[7] *Department of Photonics, National Cheng Kung University, Tainan, Taiwan, ROC*

[8] *Academy of Innovative Semiconductor and Sustainable Manufacturing, National Cheng Kung University, Tainan, Taiwan, ROC*

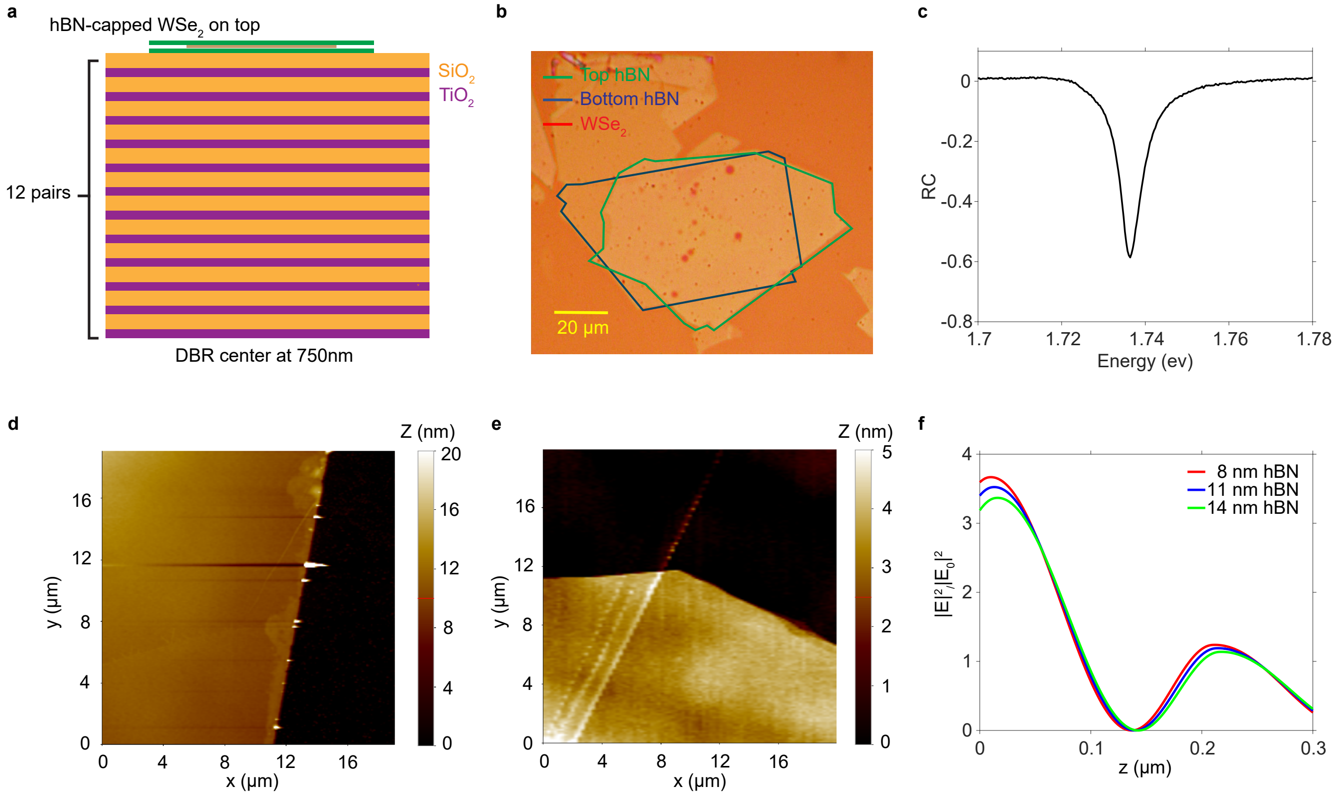


FIG. S1: The bare DBR-monolayer device. **a-b,** Schematic and optical image of the device with an hBN-encapsulated $WSe_2$ monolayer (ML) placed on top of a $SiO_2/TiO_2$ distributed Bragg reflector (DBR). **c,** Measured $WSe_2$ ML reflectance contrast. **d-e,** AFM results for bottom **(d)** and top **(e)** hBN. The hBN thickness and variance are obtained by comparing the height of hBN area vs. $SiO_2$ area. The thicknesses of the bottom and top hBN layers are $11 \pm 3$ nm and $4 \pm 1$ nm, respectively. **f,** Transfer matrix simulation of field distribution in the 2D material and DBR for different bottom hBN thickness. The TMD is at Z=0, where the field enhancement is 3.4 for bottom hBN thickness of 11 nm. The top hBN layer has a fixed thickness of 4 nm as it has less impact on the field enhancement at the ML.

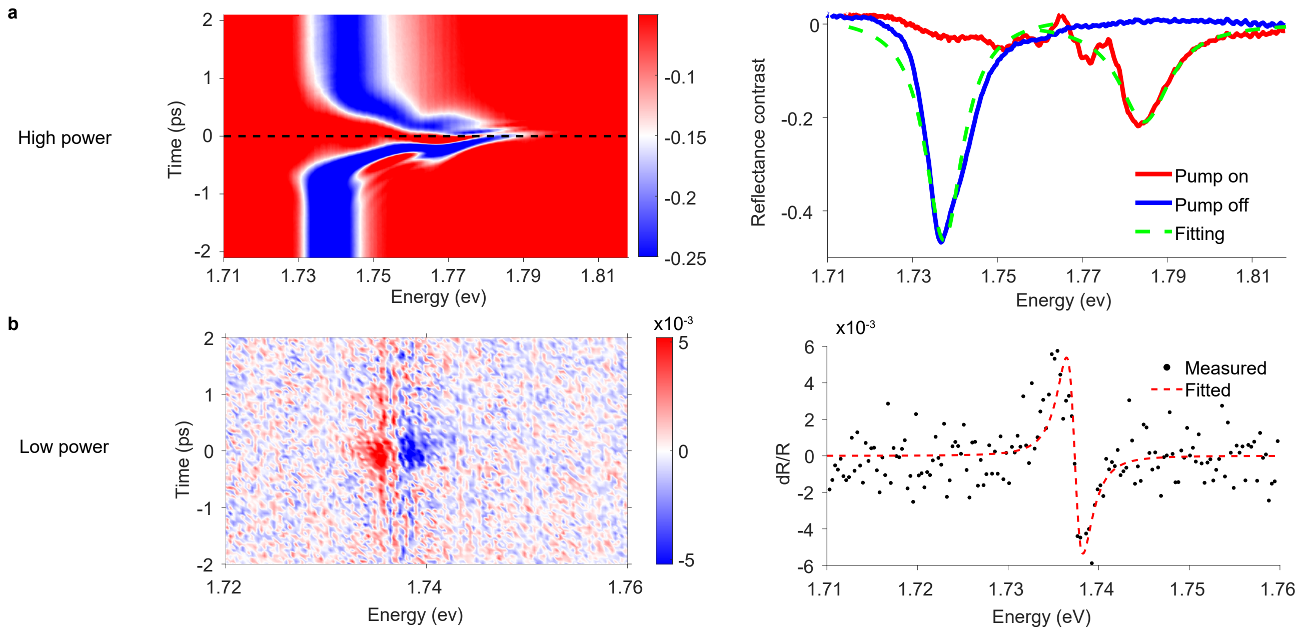


FIG. S2: Examples of fitting of the exciton shift at zero time delay. (a) A high-field large shift example. The left panel shows the co-circularly polarized reflection spectra $R_{++}(t)$ at different probe delay time $t$ relative to the pump for pump fluence 1200 fJ/$\mu$m$^2$. The black dashed line marks the zero time delay. The right panel shows the corresponding zero time delay reflection spectra with pump-on (red) and pump-off (blue), each fitted by a Lorentzian function (green dashed lines). (b) A weak-field small shift example. Left panel shows co-circular differential reflectance spectra $\Delta R_{++}(t)/R_{++}(t)$ at different probe delay with a pump fluence of 0.12 fJ/$\mu$m$^2$. The right panel shows the corresponding spectrum (black dots) fitted by the differential of two Lorentzian functions (red dashed line). In both (a) and (b), the energy difference between the two Lorentzian functions give the shifts of the exciton resonance.

# S1. SATURATION OF THE CAVITY-ENHANCED OSE

## A. Saturation due to OSE-induced cavity shift

The optical Stark shift of the exciton resonance may lead to a shift in the cavity resonance, which in turn affects the OSE. Here we analyze this effect.

We consider a half-wavelength Fabry-Pérot (FP) cavity made of two mirrors of reflectance $R$, with cavity length $l/2$ and cavity resonance $l$. The quality factor of the cavity is defined as $Q = 2\pi \frac{E_{tol}}{\Delta E_{tol}}$, where $E_{tol}$ represents the total stored energy in the cavity, and $\Delta E_{tol}$ is the energy loss per cycle of light, given by $\Delta E_{tol} = (1 - R^2)E_{tol}$. We have $Q = \frac{2\pi}{1-R^2}$ and $R(Q) = \sqrt{1 - \frac{2\pi}{Q}}$.

The cavity enhancement factor for a monochromatic light of wavelength $\lambda$ is $\hat{\eta}_{cav}(Q, \lambda, l) = \frac{1-R(Q)}{(1-R(Q))^2+4R(Q)\cdot sin^2(\phi(\lambda,l))}$ [1], whre $2\phi$ is the round-trip phase change of light in the cavity, given by $\phi(\lambda, l) = \pi\frac{l}{\lambda}$. For a Gaussian pulse typically used in experiments, its line shape can be described by $I_{beam}(\lambda, \lambda_0, w_0) = \exp(-\frac{(\lambda-\lambda_0)^2}{2(w_0/2.355)^2})/(\sqrt{2\pi}w_0/2.355)$, where $\lambda_0$ and $w_0$ is the center wavelength and linewidth of the pulse, respectively. The integration of $I_{beam}$ over $\lambda$ is normalized to $\equiv 1$. We estimate the enhancement factor of the pulse by averaging over the wavelength:

$$\bar{\eta}_{cav}(Q_0, w_0, \lambda_0, l_0) = \int_{-\infty}^{\infty} \hat{\eta}_{cav}(Q_0, \lambda, l_0) \cdot I_{beam}(\lambda, \lambda_0, w_0) d\lambda. \tag{S1}$$

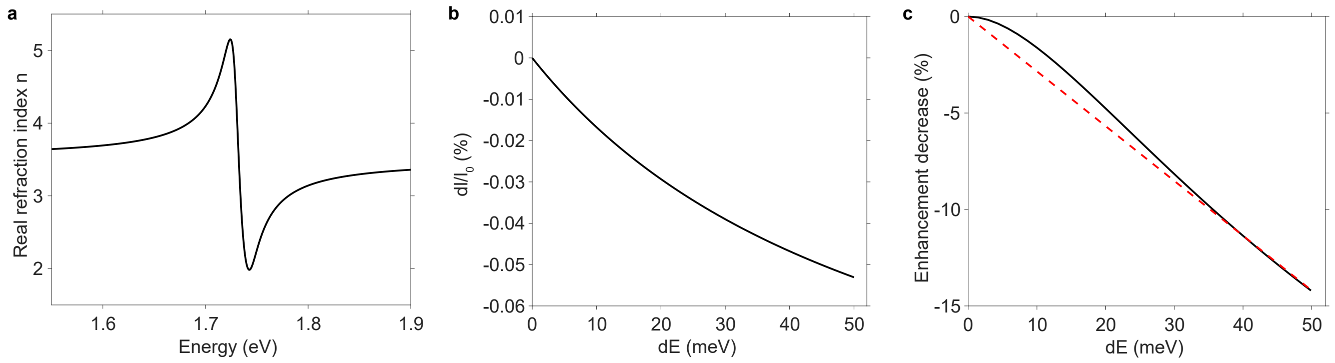


FIG. S3: Effects of the shift of the cavity resonance due to the giant cavity enhanced OSE. **a,** Real refractive index of TMD ML used in our simulation. **b,** Relative change of the cavity optical path length versus the optical Stark shift of the exciton resonance. **c,** Relative change of the enhancement factor, $\delta\eta_{cav}/\eta_{cav}$, versus the optical Stark shift of the exciton resonance (black curve). The red dashed line is a linear approximation.

The TMD monolayer can be modeled as a dielectric layer inside the cavity with a thickness $t = 1$ nm, a constant background permittivity $\chi_B = 3.5^2$, and an exciton resonance represented by a Lorentz oscillator with permittivity $\chi(E) = \frac{f_0}{E_0^2 - E^2 - iE\gamma}$. Here $f_0$ is the oscillator strength. $E_0$ is the exciton resonance. $\gamma$ is the exciton linewidth. We use $f_0 = 0.54$ eV$^2$, $E_0 = 1.74$ eV, and $\gamma = 0.01$ eV to simulate our current system (Fig. 1c grey dashed line). The real and imaginary refractive indices are $n + ik = \sqrt{\chi_B + \chi}$, and $n(\lambda)$ around the exciton resonance is shown in (Fig. S3a). When OSE leads to a shift of the exciton resonance by $\Delta E$, the dispersion of $n(\lambda)$ is shifted correspondingly, resulting in a change in $n$ by: $dn(\Delta E) = n\left(\frac{hc}{l_0} - \Delta E\right) - n\left(\frac{hc}{l_0}\right)$, which leads to a shift of the cavity resonance by $dl(\Delta E) = 2tdn(\Delta E)$. The shifted cavity resonance is $l = l_0 + dl(\Delta E)$ (Fig. S3b), which in turn results in a modified cavity enhancement factor that now also depends on $\Delta E$: $\bar{\eta}_{cav} = \bar{\eta}_{cav}(Q_0, w_0, \lambda_0, l_0, \Delta E)$. The modified $\bar{\eta}_{cav}$ in turn leads to a change in $\Delta E$. We iteratively compute $\bar{\eta}_{cav}$ and $\Delta E$, until the change in $\bar{\eta}_{cav}$ due to change in the previous iteration of $\Delta E$ is small than 0.1%.

Fig. S3c shows the results of the calculated decrease of enhancement factor $\bar{\eta}_{cav}$ with exciton shift $\Delta E$ for our cavity. At the maximum pump intensity of $I_{max} = 0.33$ GW/cm$^2$, we measured $\Delta E = 46$ meV, corresponding to a decrease of $\bar{\eta}_{cav}$ by 13%. As a simplified estimate, we approximate the reduced enhancement as $\eta_{cav}(1 - \beta I)$, with $\beta = \frac{13\%}{0.33 \text{ GW/cm}^2}$ $\approx 0.39$ (GW/cm$^2$)$^{-1}$ (red dashed line in Fig. S3c). Using the linear approximation, we modify the optical Stark shift given by Eq. 2 as:

$$\Delta E = -\Delta + \sqrt{\Delta^2 + 4\mu_{gX}^2 \eta_{cav}(1 - \beta I)|\varepsilon_{in}|^2} = -\Delta + \sqrt{\Delta^2 + 4\frac{\mu_{gX}^2}{\frac{1}{2}c\epsilon_0}\eta_{cav}(1 - \beta I)I}. \quad \text{(S2)}$$

Here $I$ is the input beam intensity: $I = \frac{1}{2}c\epsilon_0|\varepsilon_{in}|^2$, $c$ is the speed of light. The modified $\Delta E(I)$ is shown by the dashed blue line in Fig. 2g.

Note that the cavity resonance only changed by about 0.4 nm, or about 1 meV, at the maximum $\Delta E$ measured. So one can shift the pump wavelength to follow the shift of the cavity resonance, with negligible change to the detuning and recovering the original $\bar{\eta}_{cav}$.

### B. Saturation due to pump induced excitations

The red-detuned Gaussian pump may still generate excitations in the TMD monolayer due to linear and two photon absorption (TPA).

Fig. S4a presents a normalized photoluminescence (PL) spectrum with the red-detuned pump intensity = 0.12 GW/cm$^2$. From the spectrum, we can clearly resolve three features: exciton (1.74 eV), biexciton (1.72 eV), and trion (1.71 eV).

Fig. S4b presents the intensity dependence of integrated PL counts as varying excitation power in Fig. S4a. The dependence is fitted very well by a second-order polynomial:

$$\frac{N(I)}{N_0} = (5.6 \pm 1.2)I^2 + (1.1 \pm 0.4)I. \tag{S3}$$

with fitted $a_1 = 1.1 \pm 0.4$ (GW/cm$^2$)$^{-1}$ and $a_2 = 5.6 \pm 1.2$ (GW/cm$^2$)$^{-2}$. Here $N_0$ is the population of excitations at the maximum pump intensity used. The result shows that linear absorption and TPA are the main contributions. Above $P \sim 0.2$ GW/cm$^2$, TPA dominates.

Including both saturation of the oscillator strength due to the excitations and the cavity shift, we obtain:

$$\Delta E = -\Delta + \sqrt{\Delta^2 + 4\frac{\mu_{gX}^2}{\frac{1}{2}c\epsilon_0}\eta_{cav}(\frac{1}{1+\alpha\frac{N(I)}{N_0}})(1-\beta I)I} \tag{S4}$$

Equation. S3- S4 fit the measured $\Delta E(I)$ versus $I$ very well with $\alpha = 1.54 \pm 0.35$ as the only fitting parameter (red line in Fig. 2g). Fixed parameters are $\Delta = 67$ meV, $\mu_{gX}^2 \cdot \eta_{cav} = 4942$ Debye$^2$, and $\beta = 0.39$ (GW/cm$^2$)$^{-1}$.

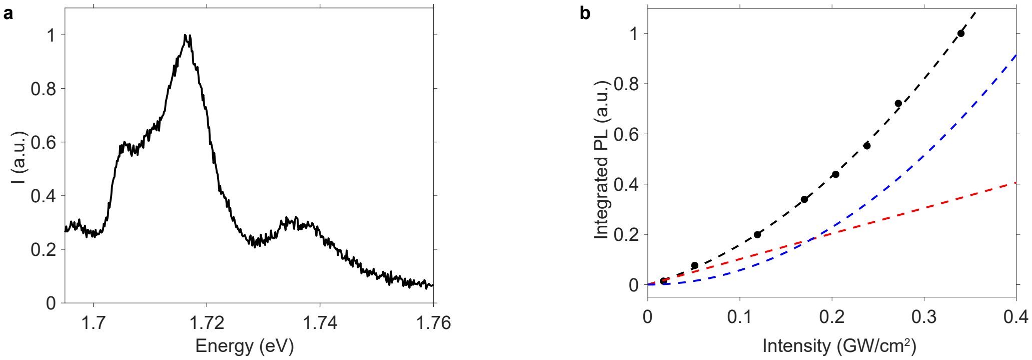


FIG. S4: Real excitations generated by the pump with 67 meV red detuning. **a,** An example of the photoluminescence spectrum at 0.12 GW/cm$^2$ pump intensity, showing peaks at the exciton (1.74 eV), biexciton (1.72 eV), and trion (1.71 eV) resonances. **b,** The dependence of the measured time-integrated PL counts on pump intensity (black dots) fitted by a second-order polynomial (black curve). The red and blue curves represent the fitted linear and quadratic components, respectively.

## C. Maximum OSE shift

The maximum exciton shift of about 46 meV in our device is mainly limited by saturation of the exciton oscillator strength as a result of absorption of the pump. Linear absorption is due to the finite linewidth of the exciton resonance and the pump pulse. Our exciton resonance is inhomogeneously broadened. Here we consider the effect of a narrower exciton linewidth.

According to Beer's law, light absorption follows an exponential attenuation: $\delta I = (1 - e^{-\frac{2\pi}{\lambda}\cdot 2kz})I_0$, where $\delta I$ represents the decreased intensity related to absorption, $k$ is the imaginary refractive index, $\lambda$ is the light wavelength. Due to the nanoscale thickness $z$ of the 2D material, we can simplify the expression:

$$\delta I \approx I_0 \frac{2\pi}{\lambda} \cdot 2kz \Rightarrow \delta I \propto k \tag{S5}$$

When the input beam has a certain linewidth, the light absorption follows an average $k$ value, $k'$, based on the beam profile $I$:

$$\delta I \propto k'(E_0, \omega_0) = \frac{\int_{-\infty}^{\infty} k(E) \cdot I(E, E_0, \omega_0) dE}{\int_{-\infty}^{\infty} I(E, E_0, \omega_0) dE} \tag{S6}$$

$E_0$, $\omega_0$ are the center frequency and linewidth of the input beam. Here we consider the input beam is a Gaussian pulse with 2.3 meV (1 nm) linewidth, scanning different center frequency. Then we get the averaged $k'$.

Assuming the generation of real carriers is linearly related to absorption, we can extrapolate the linear excitation efficiency $a_1(k(\Delta))$ as a function of imaginary refractive index $k$ with pump detunings $\Delta$:

$$\frac{a_1(\Delta)}{a_{10}} = \frac{k'(\Delta)}{k'_0} \tag{S7}$$

Here, $k'_0$ denotes the imaginary refractive index of $WSe_2$ at $\Delta_0 = 67$ meV in our cavity-enhanced device, where we measured $a_{10}$ as 1.1 $(\mathrm{GW/cm^2})^{-1}$. $k'(\Delta)$ denotes the imaginary refractive index with new pump detunings $\Delta$. $k'$ is determined by Lorentz oscillator with permittivity $\chi(E) = \frac{f_0}{E_0^2 - E^2 - iE\gamma}$. Then we are able to extrapolate overall excitation efficiency:

$$\frac{N(I, k(\Delta))}{N_0} = a_2 \cdot I^2 + a_1(k(\Delta)) \cdot I \tag{S8}$$

Here, $a_2$ denotes the two-photon excitation efficiency, which is independent of pump detunings. $a_1(k(\Delta))$ represents the tunable linear excitation efficiency. By inserting Eq. S8 into Eq. S4 and disregarding the cavity shift effect, which can be offset by a slight adjustment in the pump frequency, we arrive at:

$$\Delta E = -\Delta + \sqrt{\Delta^2 + 4\frac{\mu_{gX}^2}{\frac{1}{2}c\epsilon_0}\eta_{cav}(\frac{1}{1+\alpha\frac{N(I,k(\Delta))}{N_0}})I} \tag{S9}$$

From Eq. S9, the maximum shift, $\Delta E_{max}$, observed when varying the pump intensity, $I$, is given by:

$$\Delta E_{max} = -\Delta + \sqrt{\Delta^2 + 4\frac{\mu_{gX}^2}{\frac{1}{2}c\epsilon_0}\eta_{cav}(\frac{1}{2\sqrt{\alpha a_2} + \alpha a_1(k(\Delta))})} \tag{S10}$$

Here $\Delta E$ may be increased by decreasing the exciton linewidth, which is ultimately limited by the radiative decay rate, or $\mu_{gX}$. Given exciton linewidth, the exciton shift increases with increasing the field intensity until saturation overwhelms, leading to a "saturated" shift. The linear absorption can be decreased by increasing $\Delta$, although the shift scales inverse proportionally with $\Delta$. This gives us an optimal detuning for maximal exciton shift.

Using Eq. S10, We calculate the "saturated" shift versus pump intensity for each detuning, limited by saturation, and then calcuated this shift versus the detuning. The results are shown in Fig. 4 for our device, for a device with an exciton medium of a linewidth of 1 meV, close to the radiative limit, and a device with 1 meV exciton linewidth and 10-fold smaller TPA coefficient $a_2$.

## S2. CROSS-CIRCULAR CAVITY-EXCITONIC OPTICAL STARK EFFECT

When measuring the cross-polarized optical Stark effect (OSE), we observe a distinct exciton redshift. This phenomenon has been attributed to many-body Coulomb interactions, with biexcitons being the primary contributors [2–4]. In our case, when the red-detuning of the pump, $\Delta E = 67$ meV, exceeds the biexciton binding energy $E_b$, the Floquet effect involving the biexciton state $|X_+X_-\rangle$ and the $K_-$ valley exciton state $|X_-\rangle$ can be described as follows:

$$H = \begin{pmatrix} 0 & \mu_{gX}|\varepsilon| & 0 & 0 \\ \mu_{gX}|\varepsilon| & \Delta & 0 & 0 \\ 0 & 0 & E_X & \mu_{gX}|\varepsilon| \\ 0 & 0 & \mu_{gX}|\varepsilon| & E_X - E_b + \Delta \end{pmatrix}. \tag{S11}$$

Diagonalizing the Hamiltonian gives the energy shift for the $|X_-\rangle$ state:

$$\Delta E_- = \frac{1}{2}(-E_b + \sqrt{\Delta^2 + 4\mu_{gX}^2|\varepsilon|^2} - \sqrt{(\Delta - E_b)^2 + 4\mu_{gX}^2|\varepsilon|^2}) \tag{S12}$$

Furthermore, the saturation effects discussed in previous Supplement also apply here:

$$\mu_{gX}^2|\varepsilon|^2 \to \frac{\mu_{gX}^2}{\frac{1}{2}c\epsilon_0}\eta_{cav}(\frac{1}{1+\alpha\frac{N(I)}{N_0}})(1-\beta I)I. \tag{S13}$$

By combining Eqs. S4, S12, and S13, we can predict the valley splitting as a function of the pump intensity by fitting the biexciton binding energy, $E_b$. The result is shown in Fig. 2h, with a fitted value of $E_b = 19 \pm 13$ meV.

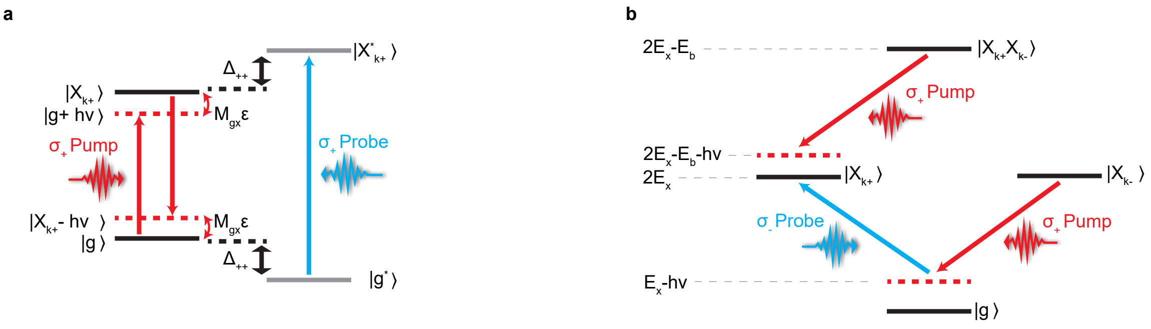


FIG. S5: Illustration of the red-detuned chiral OSE. **a,** Co-circular OSE schematic: pump the $K_+$ valley and probe the $K_+$ valley. **b,** Cross-circular OSE schematic: pump the $K_+$ valley and probe the $K_-$ valley.

[1] N. Ismail, C. C. Kores, D. Geskus, and M. Pollnau, Fabry–pérot resonator: spectral line shapes, generic and related airy distributions, linewidths, finesses, and performance at low or frequency-dependent reflectivity, Opt. Express **24**, 16366 (2016).

[2] M. Combescot and R. Combescot, Excitonic Stark Shift: A Coupling to "Semivirtual" Biexcitons, Physical Review Letters **61**, 117 (1988).

[3] E. J. Sie, C. H. Lui, Y.-H. Lee, J. Kong, and N. Gedik, Observation of Intervalley Biexcitonic Optical Stark Effect in Monolayer $WS_2$, Nano Letters **16**, 7421 (2016).

[4] C.-K. Yong, J. Horng, Y. Shen, H. Cai, A. Wang, C.-S. Yang, C.-K. Lin, S. Zhao, K. Watanabe, T. Taniguchi, S. Tongay, and F. Wang, Biexcitonic optical Stark effects in monolayer molybdenum diselenide, Nature Physics **14**, 1092 (2018).
